# Effect of tranexamic acid on planktonic and biofilm susceptibility of *Candida albicans*

**DOI:** 10.1186/s12866-026-05235-w

**Published:** 2026-05-30

**Authors:** Liqin Yao, Rui Liu, Qiangde Hu, Yicheng Li, Boyong Xu, Xiaogang Zhang, Li Cao, Wenbo Mu

**Affiliations:** Department of Orthopaedics, First Affiliated Hospital of Xinjiang Medical University, Urumqi, Xinjiang 830054 China

**Keywords:** Tranexamic acid, *Candida albicans*, Biofilm, Fluconazole, Voriconazole, Caspofungin, Amphotericin B

## Abstract

**Background:**

Fungal periprosthetic joint infection remains a challenging complication in revision arthroplasty. *Candida albicans* (*C. albicans*) is among the most frequently reported fungal pathogens, and biofilm formation further limits antifungal efficacy. Tranexamic acid (TXA) is routinely used to reduce perioperative blood loss in arthroplasty and may come into direct contact with antifungal agents within the joint environment. However, whether TXA modifies antifungal activity against *C. albicans* remains unclear. This study aimed to evaluate the effect of TXA on the in vitro activity of representative antifungal agents against *C. albicans* under planktonic and established biofilm conditions.

**Results:**

Baseline planktonic MICs for FLC, VRC, CAS, and AMB were 0.75, 0.5, 1, and 0.75 µg/mL, respectively. In the presence of TXA, susceptibility shifts were class-dependent: MICs for VRC and CAS decreased four-fold and two-fold, respectively, while AMB exhibited a two-fold increase and FLC remained unchanged. Established biofilms showed markedly reduced antifungal susceptibility, and TXA further altered the XTT-derived biofilm metabolic inhibition profiles. For FLC, TXA increased peak metabolic inhibition from approximately 50–55% in saline to 85–90%, with an XTT-derived MBEC₉₀ of 192 µg/mL. In contrast, TXA attenuated VRC-mediated biofilm metabolic inhibition, producing a right-shifted dose-response profile relative to saline conditions. For CAS, TXA lowered the XTT-derived activity threshold from 256 to 128 µg/mL, although the response curves converged at higher concentrations. For AMB, TXA reduced apparent biofilm activity, increasing the XTT-derived minimum biofilm eradication concentrations (MBECs) threshold from 1.5 to 12 µg/mL.

**Conclusions:**

TXA differentially modulated the apparent in vitro activity of representative antifungal agents against *C. albicans* in an antifungal class- and growth-state-dependent manner. The discordance between planktonic and antibiofilm responses under TXA co-exposure supports further mechanistic and translational validation in clinically relevant *Candida* biofilm models.

## Introduction

Periprosthetic joint infection (PJI) remains a devastating complication after arthroplasty, associated with substantial morbidity and revision burden [1–3]. Fungal PJI accounts for less than 1% of reported cases, yet outcomes are disproportionately poor [4]. Reported failure rates frequently exceed 40%, and mortality has been described in the range of 25–40% [5]. *Candida albicans* (*C. albicans*) is the predominant fungal pathogen in these cases. Its robust biofilm-forming capacity confers antifungal tolerance, often leading to recurrent or persistent infections [6]. These observations indicate a narrow therapeutic margin and highlight the need to define perioperative factors that could influence antifungal effectiveness in this setting.

Management commonly combines revision surgery with antifungal therapy [7]. Surgery provides source control [8]. It removes infected tissue and contaminated implants, enables thorough debridement, and reduces the microbial burden to a level more amenable to pharmacologic suppression. Antifungal therapy is then continued for a prolonged course using agents drawn from major antifungal classes [9, 10]. Standard susceptibility testing is performed on planktonic isolates, but implant-associated infection often involves established surface-adherent communities. Under these conditions, antifungal activity may be reduced compared with planktonic growth.

Revision arthroplasty is associated with substantial perioperative blood loss and increased transfusion requirements compared with primary procedures [11]. Because transfusion exposure has been linked to postoperative morbidity in arthroplasty populations, blood-conservation strategies are routinely incorporated into revision pathways. Tranexamic acid (TXA) is a synthetic lysine analogue with amino and carboxyl functional groups. Clinically, it acts as an antifibrinolytic agent by competitively inhibiting the interaction between plasminogen and fibrin, thereby reducing fibrinolysis and perioperative blood loss. TXA is widely used to reduce bleeding and transfusion exposure in hip and knee arthroplasty, including revision settings [12, 13]. It has been reported to enhance the activity of certain antibiotics such as vancomycin and gentamicin [14, 15]. In fungal PJI, TXA may be present at high concentrations within the perioperative joint environment and may come into direct contact with antifungal agents [16].

Whether TXA alters antifungal activity remains unknown. Therefore, this in vitro study evaluated whether TXA modifies the activity of representative antifungal agents from major classes, namely fluconazole (FLC), voriconazole (VRC), caspofungin (CAS), and amphotericin B (AMB), against *C. albicans* under planktonic and established biofilm conditions.

## Methods

**Fig. 1:**
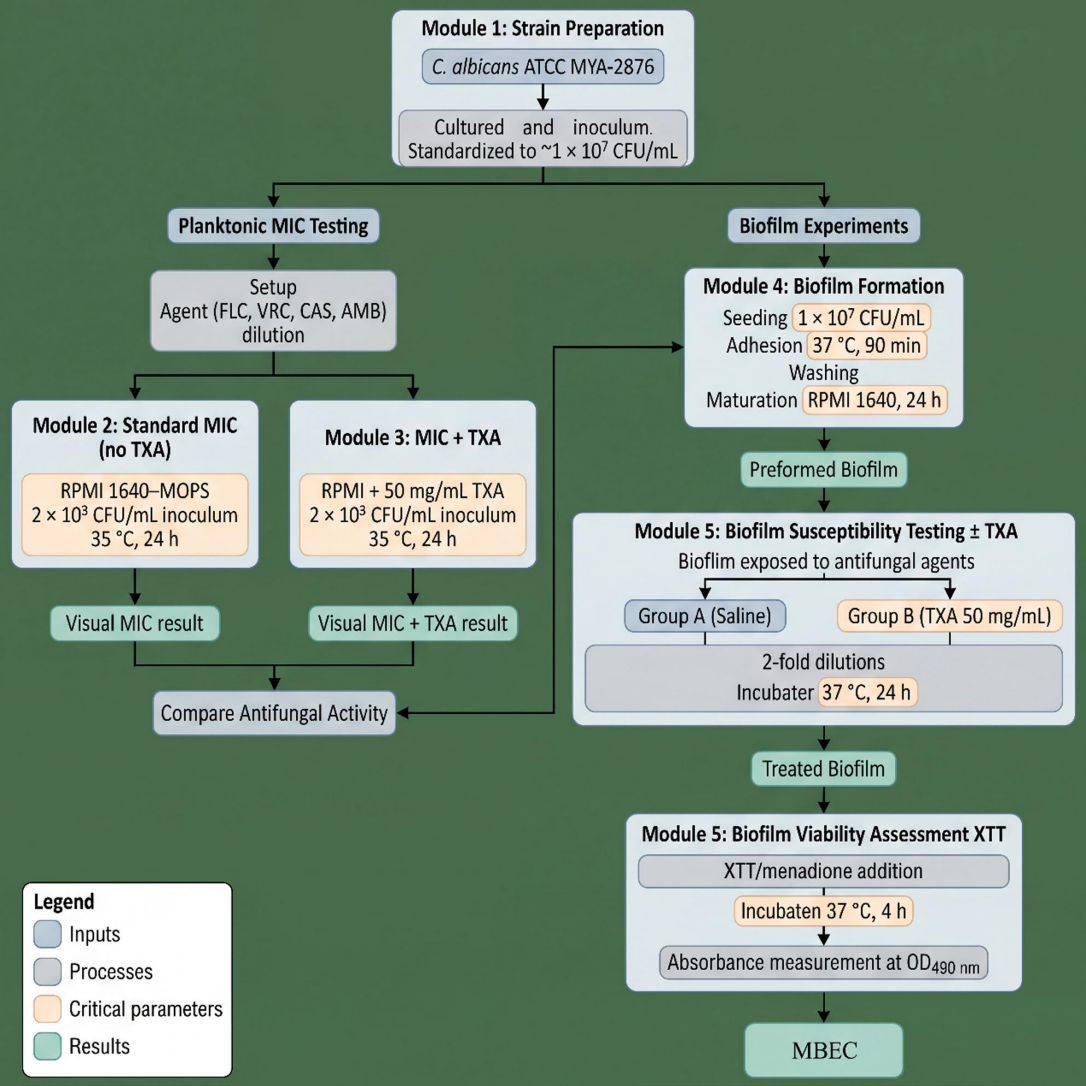
Experimental workflow for assessing TXA-antifungal interactions against *C. albicans* under planktonic and established biofilm conditions. Representative antifungal agents from the azole (FLC, VRC), echinocandin (CAS), and polyene (AMB) classes were tested in parallel with or without TXA. Planktonic susceptibility was determined by broth microdilution (MIC), and MBECs was quantified using the XTT reduction assay (metabolic inhibition relative to the corresponding drug-free control)

An overview of the experimental workflow is provided in Fig. 1.

### Strain and culture conditions

*C. albicans* (ATCC MYA-2876) was obtained from the American Type Culture Collection and used as the reference strain in this study. For broth culture, the yeast was incubated on yeast extract peptone dextrose (YPD) media (Solario, Beijing, China). For solid culture, it was grown on sabourauds dextrose agar (SDA, Hopebio, Qingdao, China) plate for 24 h. Fungal growth was monitored by measuring the optical density at 600 nm (OD600) using a SpectraMax multimode microplate reader (Molecular Devices Ltd., San Jose, CA, USA). The suspension was adjusted to approximately 1 × 10^7^ colony-forming units (CFU)/mL.

### Planktonic susceptibility testing

In vitro antifungal susceptibility was evaluated using a broth microdilution assay to determine the minimum inhibitory concentrations (MICs) of representative agents against planktonic *C. albicans*. Following CLSI M27-A3 guidelines [17], four antifungal agents were tested: azoles (FLC and VRC), an echinocandin (CAS), and a polyene (AMB). RPMI 1640 medium buffered with 0.165 mol/L MOPS at pH 7.0 was used as the test medium. Drug stock solutions were prepared according to solubility, with 1% DMSO used as a co-solvent when required. These stocks were diluted in RPMI 1640-MOPS to generate working solutions and then subjected to twofold serial dilution to produce a 2× concentration range of 0.03125–64 µg/mL. Aliquots of 100 µL were dispensed into sterile, flat-bottom 96-well microplates. Yeast-form inocula were prepared from fresh cultures and standardized to a 2× concentration. Equal volumes of inoculum and drug dilution were combined in each well to achieve a final volume of 200 µL, a final inoculum of 2 × 10³ CFU/mL, and final drug concentrations of 0.015625–32 µg/mL. Growth controls (inoculated medium without drug) and sterility controls (medium alone) were included in each plate. When DMSO was used, its final concentration was kept constant across all wells, including the corresponding solvent controls. Plates were incubated statically under aerobic conditions at 35 °C for 24 h.

To evaluate whether TXA modified planktonic antifungal susceptibility, antifungal working solutions were prepared in TXA-containing medium so that the final TXA concentration after mixing with the fungal inoculum was 50 mg/mL [18–20]. Briefly, 100 µL of the standardized fungal suspension was combined with 100 µL of the antifungal-TXA dilutions, yielding the same final antifungal concentration range (0.015625–32 µg/mL) as the standard assay. Plates were incubated and evaluated under the same conditions described above.

MIC values were determined by visual inspection, with the endpoint defined as complete inhibition of visible growth. All experiments were performed in at least three independent biological replicates.

### Biofilm susceptibility testing

*C. albicans* biofilms were developed in sterile, flat-bottom 96-well polypropylene microplates using a two-stage process consisting of adhesion and maturation. For the adhesion phase, standardized inocula of blastospores 1 × 10⁷ CFU/mL were prepared in RPMI 1640 and dispensed into each well (200 µL/well). The plates were incubated at 37℃ with orbital shaking at 75 rpm for 90 min to facilitate initial cellular attachment [21]. Following incubation, non-adherent cells were removed by careful aspiration of the supernatant, and the wells were gently washed three times with 200 µL of sterile distilled water. For the maturation phase, 200 µL of fresh RPMI 1640 was added to each well, and the plates were incubated at 37℃ with shaking (75 rpm) for 24 h. After maturation, the resulting biofilms were rinsed three times with sterile PBS (200 µL/well) to eliminate residual planktonic cells.

To evaluate the influence of TXA on the antibiofilm efficacy of FLC, VRC, CAS, and AMB, two distinct sets of antifungal concentration gradients were prepared to address the robust biofilm-forming capacity of *C. albicans*. For the standard control group, antifungal working solutions were prepared by diluting the agents in sterile normal saline. For the experimental group, the antifungal agents were dissolved and diluted directly in a TXA solution. In both groups, twofold serial dilutions were performed to achieve final antifungal concentrations ranging from 0.5× to 2048× the isolate-specific planktonic MIC. The final concentration of TXA in the experimental group was maintained at 50 mg/mL. Preformed biofilms were exposed to 200 µL of these respective treatment solutions and incubated at 37 °C for 24 h. To ensure experimental rigor and control for dilution effects, the final antifungal concentrations were identical across both the saline-based and TXA-based groups. Experimental controls included: (1) Saline-based growth controls (drug-free medium supplemented with saline). (2) TXA-supplemented growth controls (drug-free medium supplemented with 50 mg/mL TXA). (3) Sterility controls (medium containing saline or TXA without fungal inoculation).

The XTT reduction assay was used to quantify the metabolic activity of established *C. albicans* biofilms after antifungal exposure. Briefly, a 10 mM menadione stock solution was prepared in acetone and added to XTT solution at 0.5 mg/mL to obtain a final menadione concentration of 1 µM. Each well, including control wells, received 100 µL of the XTT/menadione working solution and was incubated in the dark at 37 °C for 4 h. After incubation, 70 µL of the supernatant from each well was transferred to a new microplate, and absorbance was measured at 490 nm using a microplate reader. The absorbance values were used to calculate the percentage of biofilm metabolic inhibition relative to the corresponding drug-free control.

In this study, minimum biofilm eradication concentrations (MBECs) values were derived from XTT-based metabolic inhibition data. Therefore, these values should be interpreted as metabolic inhibition thresholds rather than culture-confirmed sterilization endpoints or direct evidence of complete biofilm eradication. All experimental conditions were tested in triplicate across three independent biological assays to ensure reproducibility.

### Statistical analysis

All experiments were performed in at least three independent biological replicates, with technical triplicates included where applicable. MIC and XTT-derived MBEC values were determined from twofold serial dilution assays and are reported descriptively as endpoint-based measurements.

## Results

### Effect of TXA on planktonic susceptibility

**Fig. 2:**
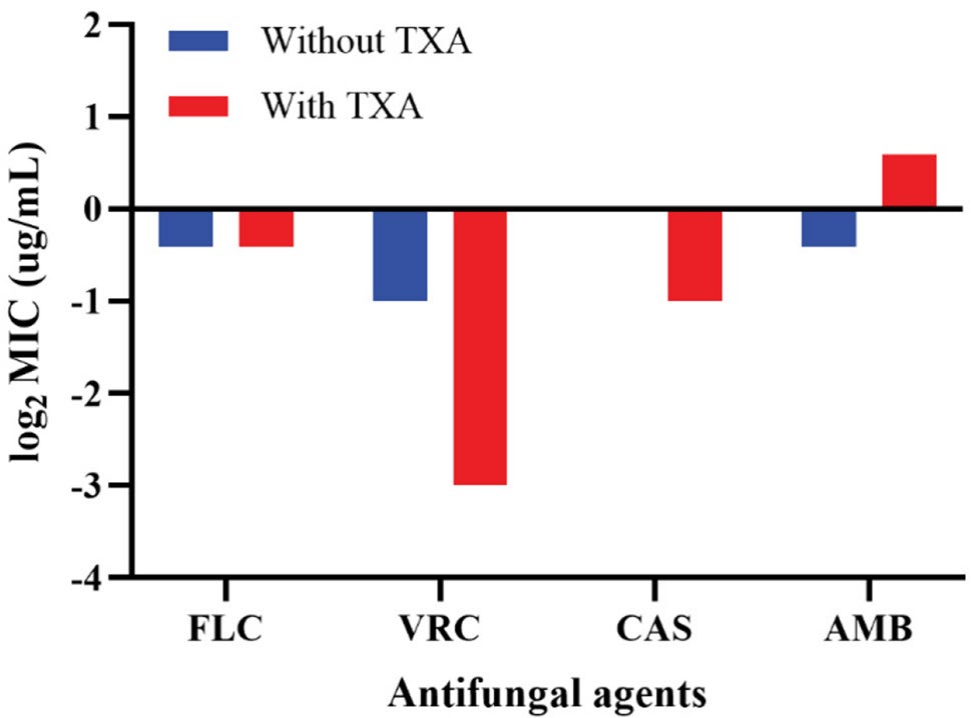
Effect of TXA on planktonic antifungal susceptibility of *C. albicans*. MICs of FLC, VRC, CAS, and AMB were determined by broth microdilution in standard medium and in the presence of TXA. The figure summarizes agent-specific MIC shifts associated with TXA exposure

In the absence of TXA, the MICs against planktonic *C. albicans* were 0.75 µg/mL for FLC, 0.5 µg/mL for VRC, 1 µg/mL for CAS, and 0.75 µg/mL for AMB. To evaluate the influence of TXA on these values, susceptibility was assessed in the presence of TXA. As shown in Fig. 2, TXA exposure resulted in agent-specific changes in MIC values. The MIC of FLC remained unchanged compared with the baseline condition. In contrast, VRC and CAS showed increased apparent activity in the presence of TXA, with fourfold and twofold reductions in MIC, respectively. By comparison, AMB exhibited a twofold increase in MIC under TXA exposure. These findings indicate that TXA differentially modified planktonic antifungal susceptibility depending on the antifungal agent tested.

### Effect of TXA on antifungal activity against established biofilms

**Fig. 3:**
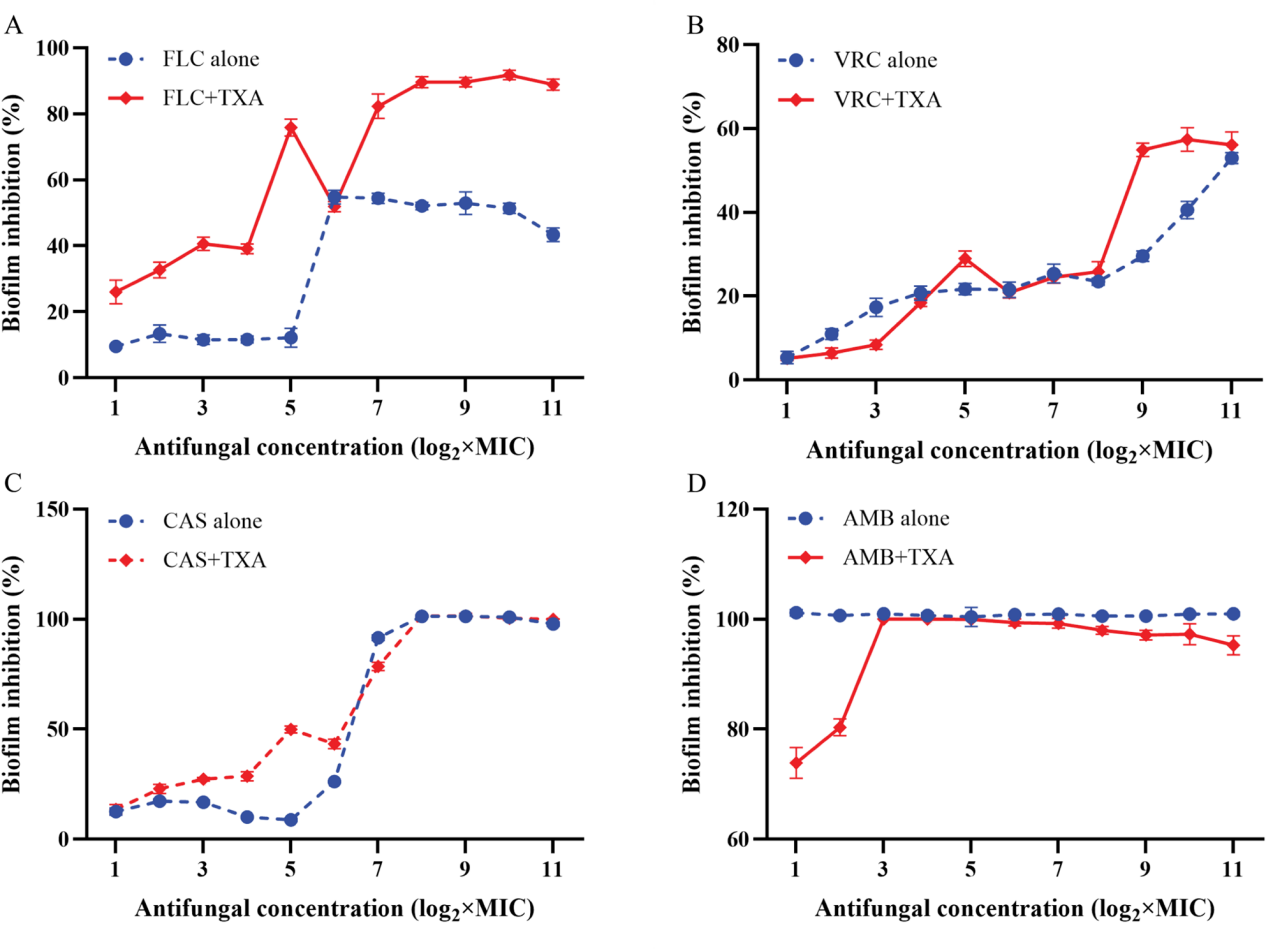
Dose-response activity of antifungal agents against established C. albicans biofilms in saline versus TXA-containing vehicle. Preformed C. albicans biofilms were exposed to twofold serial dilutions of FLC, VRC, CAS, or AMB, ranging from 0.5× to 2048× the corresponding planktonic MIC, prepared in either saline or TXA solution. **A** Dose-response inhibition curve for FLC alone and FLC combined with TXA. **B** Dose-response inhibition curve for VRC alone and VRC combined with TXA. **C** Dose-response inhibition curve for CAS alone and CAS combined with TXA. **D** Dose-response inhibition curve for AMB alone and AMB combined with TXA. Biofilm inhibition was assessed using the XTT reduction assay at OD490 and expressed as the percentage of inhibition relative to the corresponding drug-free control, namely saline alone or TXA alone. MBEC values were derived from XTT-based metabolic inhibition data and should therefore be interpreted as metabolic inhibition thresholds rather than sterilization endpoints

The activity of FLC, VRC, CAS, and AMB against established *C. albicans* biofilms was evaluated using the XTT reduction assay. In saline-based conditions, FLC and VRC showed limited metabolic inhibition of established biofilms, whereas CAS and AMB achieved higher levels of metabolic suppression within specific concentration ranges. The presence of TXA altered these dose-response profiles in an antifungal agent-dependent manner (Fig. 3).

#### Azoles

FLC and VRC showed distinct responses to TXA co-exposure. In saline, FLC produced modest biofilm metabolic inhibition, reaching a plateau of approximately 50–55%, with an XTT-derived MBEC₅₀ of 48 µg/mL. In the presence of TXA, FLC-mediated metabolic suppression increased to approximately 85–90% at higher concentrations, with an XTT-derived MBEC₉₀ of 192 µg/mL. In contrast, VRC showed reduced apparent activity under TXA-containing conditions. Although VRC in saline produced a dose-dependent increase in metabolic inhibition, with an XTT-derived MBEC₅₀ of 1024 µg/mL, TXA co-exposure resulted in a right-shifted dose-response profile, indicating that higher VRC concentrations were required to achieve comparable metabolic suppression.

#### Echinocandins

CAS demonstrated a pronounced threshold effect against established biofilms. Using the planktonic MIC (1 µg/mL) as a reference, the baseline MBEC occurred at 256 µg/mL (256× MIC). With TXA co-exposure, the MBEC decreased to 128 µg/mL (128× MIC), indicating improved activity within the low-to-mid concentration range. However, at higher concentrations where CAS alone has already achieved maximal suppression, the efficacy curves for both groups converged.

#### Polyenes

AMB exhibited the intrinsic antibiofilm activity under baseline conditions. Based on its planktonic MIC (0.75 µg/mL), the baseline MBEC was achieved at 1.5 µg/mL. Notably, the presence of TXA attenuated this potency. In the TXA-supplemented group, the concentration required to reach the MBEC increased to 12 µg/mL, representing a marked reduction in AMB effectiveness. A slight downward trend in inhibition at the highest AMB doses further underscores the interfering effect of TXA on polyene-mediated biofilm suppression.

## Discussion

TXA is widely used to reduce perioperative blood loss in revision arthroplasty, but its potential influence on antifungal activity remains insufficiently defined. In this preliminary in vitro study, we evaluated whether TXA modifies the activity of representative antifungal agents against *C. albicans* under planktonic and established biofilm conditions. Our findings indicate that TXA is not pharmacologically neutral in this experimental setting; instead, its effects vary according to both antifungal class and fungal growth state. Specifically, TXA differentially altered planktonic susceptibility and biofilm metabolic inhibition depending on the antifungal agent tested. These observations suggest that TXA-antifungal interactions may be more complex than previously assumed and warrant further mechanistic and translational validation in clinically relevant fungal PJI models.

In the planktonic phase, *C. albicans* ATCC MYA-2876 exhibited high baseline susceptibility to all four representative antifungal agents. VRC and AMB showed the greatest intrinsic activity, with MIC values of 0.5 µg/mL and 0.75 µg/mL, respectively. FLC showed a comparable MIC of 0.75 µg/mL, whereas CAS exhibited a slightly higher MIC of 1 µg/mL. These susceptibility profiles are consistent with the established mechanisms of the tested antifungal classes. AMB exerts its fungicidal activity primarily by binding to membrane ergosterol and disrupting fungal membrane integrity [22, 23]. VRC and FLC inhibit lanosterol 14α-demethylase and thereby impair ergosterol biosynthesis during active yeast growth [24, 25], whereas CAS inhibits β-1,3-D-glucan synthesis and disrupts fungal cell wall integrity [26, 27]. Together, these baseline data confirm that the selected strain was highly susceptible to conventional antifungal agents in its planktonic yeast form. Importantly, the addition of 50 mg/mL TXA induced distinct, agent-specific shifts in planktonic susceptibility, indicating that TXA was not pharmacologically neutral in this system. TXA enhanced the apparent activity of VRC and CAS, resulting in four-fold and two-fold reductions in their MIC values, respectively. In contrast, TXA did not alter the MIC of FLC but attenuated the activity of AMB, as reflected by a two-fold increase in its MIC. These divergent responses suggest that TXA does not function as a universal enhancer of antifungal activity. Rather, its influence appears to depend on the antifungal class and the biological target of each agent. The chemical and pharmacological properties of TXA may partly explain these observations. TXA is a synthetic lysine analogue and a small hydrophilic molecule containing amino and carboxyl groups [28]. At high local concentrations, TXA may alter the physicochemical microenvironment surrounding fungal cells, influence cell surface interactions, or modify local drug availability. These effects may contribute to the enhanced apparent activity of VRC and CAS during planktonic growth. Conversely, the reduced activity of AMB suggests a possible interference with polyene-mediated antifungal activity, potentially through altered drug availability, modified drug-cell surface interactions, or changes in the microenvironment required for efficient ergosterol-associated membrane disruption. These findings support and refine the emerging concept that TXA can modulate antimicrobial activity. The enhanced activity observed with VRC and CAS is consistent with previous reports showing that TXA can act synergistically with certain antibacterial agents, including vancomycin and gentamicin, against staphylococci [29, 30]. However, our results indicate a higher level of pharmacological complexity in fungal systems. Unlike the broader synergy reported in some bacterial models, TXA-antifungal interactions were strictly agent-dependent, shifting from potentiation with VRC and CAS to attenuation with AMB. Therefore, routine TXA use in revision arthroplasty should not be assumed to be uniformly compatible with all concurrent prophylactic or therapeutic regimens. Although the hemostatic benefits of TXA are well established, its divergent effects on antifungal potency support a more cautious and tailored approach when selecting antifungal agents for the perioperative management of fungal PJI.

In the biofilm phase, however, the interaction between TXA and antifungal agents became substantially more complex. A key driver of *C. albicans* pathogenicity in PJI is its ability to transition between yeast and hyphal forms, a process that promotes surface adhesion, three-dimensional biofilm maturation, and the development of a protective extracellular matrix [31]. Consistent with this biology, established *C. albicans* biofilms exhibited profound tolerance to all tested antifungal agents compared with planktonic cells. Even when antifungal concentrations were increased up to 2048-fold above their respective planktonic MICs, complete metabolic suppression of the sessile community was not uniformly achieved. Therefore, the MBEC values derived from the XTT assay should be interpreted primarily as metabolic inhibition thresholds rather than as definitive evidence of complete biofilm eradication. Within this highly tolerant biofilm environment, the addition of 50 mg/mL TXA acted as a non-uniform pharmacological modulator of antifungal activity. The most notable divergence occurred within the azole class, where TXA exerted opposite effects depending on the specific agent. For FLC, which achieved only modest metabolic suppression as monotherapy, with inhibition plateauing at approximately 50–55%, TXA co-exposure markedly enhanced its antibiofilm activity, increasing suppression to approximately 85–90% at higher concentrations. This effect may be related to TXA-mediated changes in the extracellular matrix or local physicochemical microenvironment, which could facilitate FLC penetration, increase intrabiofilm drug availability, or enhance the susceptibility of metabolically active sessile cells to azole-mediated inhibition of ergosterol biosynthesis. In contrast, VRC showed a reversed interaction pattern in the biofilm state. Although TXA potentiated VRC activity against planktonic cells, its presence attenuated the antibiofilm response of VRC, as reflected by a rightward shift of the dose-response curve. This finding indicates that TXA-antifungal interactions are not determined solely by antifungal class, but are also strongly influenced by fungal growth state and biofilm architecture. In mature biofilms, the extracellular matrix may restrict antifungal penetration, sequester drugs, alter local drug distribution, or modify access to fungal cellular targets. Accordingly, TXA may differentially affect the intrabiofilm availability or target accessibility of individual azoles, thereby enhancing FLC activity while reducing VRC potency under biofilm conditions. A similarly concentration-dependent interaction was observed with CAS. CAS alone displayed a pronounced threshold effect against established biofilms. When TXA was added, the effective activity threshold of CAS was reduced, resulting in enhanced suppression at low-to-intermediate concentrations. However, as CAS concentrations approached maximal baseline inhibition, the dose-response curves converged. This pattern suggests that TXA may facilitate early CAS activity against susceptible biofilm subpopulations or improve access to cell-wall-associated targets, but does not increase the overall efficacy ceiling of CAS once maximal inhibition is reached. Thus, TXA appears to broaden the effective activity window of CAS rather than fully overcome the intrinsic tolerance of mature *C. albicans* biofilms. The most clinically concerning interaction was observed with AMB. As the agent with the intrinsic antibiofilm activity in this study, AMB was consistently attenuated by TXA. The increase in the AMB MBEC from 1.5 µg/mL to 12 µg/mL in the presence of TXA indicates a marked loss of potency under biofilm conditions. Given that AMB exerts its antifungal effect primarily through ergosterol binding and membrane disruption, this attenuation may reflect reduced drug availability, altered drug-matrix interactions, impaired access to fungal membranes, or changes in the local microenvironment required for efficient polyene-mediated membrane damage. Because AMB remains an important therapeutic option for refractory fungal infections, this antagonistic interaction warrants particular caution in the context of fungal PJI. Collectively, these findings indicate that TXA acts as a state-dependent and agent-specific modulator of antifungal activity in *C. albicans* biofilms. Previous work by Benjumea et al. [32] suggested that TXA may provide broad antimicrobial benefit through synergy with certain antibiotics against staphylococcal biofilms. However, the present data indicate that such antimicrobial effects cannot be directly extrapolated to fungal biofilm systems. In *C. albicans*, the direction and magnitude of TXA-antifungal interactions appear to depend on antifungal class, drug concentration, and fungal growth state.

Several limitations should be acknowledged. This preliminary in vitro study used only one antifungal-susceptible *C. albicans* reference strain, ATCC MYA-2876; therefore, the findings should be interpreted as strain-specific and not broadly generalizable. Clinical *C. albicans* isolates may differ in antifungal susceptibility, biofilm-forming capacity, matrix composition, metabolic activity, and biofilm-associated tolerance. Moreover, fungal PJI may also involve non-*albicans Candida* species, including *C. parapsilosis*, *C. glabrata*, *C. tropicalis*, and the emerging multidrug-resistant species *C. auris*. Future studies should therefore include multiple clinical isolates, resistant or reduced-susceptibility strains, and clinically relevant non-*albicans Candida* species. The lack of PJI-derived clinical isolates or patient-derived samples further limits translational relevance. Although RPMI 1640 provides standardized and reproducible conditions for *Candida* susceptibility testing, it does not fully recapitulate the complex PJI microenvironment, including synovial fluid, host proteins, fibrin deposits, immune components, prosthetic material surfaces, and dynamic local drug concentrations. In addition, although 50 mg/mL TXA was selected to model a high local exposure condition relevant to topical or intra-articular use, sustained exposure at this concentration in vitro may not reflect the time-varying intra-articular pharmacokinetics in vivo. Validation in more clinically relevant systems, such as synovial fluid- or serum/plasma-supplemented media, fibrin-conditioned prosthetic surfaces, biomaterial-based biofilm models, dynamic flow systems, or ex vivo joint fluid models, is warranted. Although TXA-alone controls were included at 50 mg/mL, a full TXA concentration-response analysis was not performed. Thus, the present findings indicate TXA-associated modulation of antifungal activity under the tested conditions, but do not define the intrinsic antifungal or antibiofilm activity of TXA. Because formal synergy assays were also not conducted, the results should not be interpreted as definitive evidence of synergistic or additive interactions. Future studies should include serial TXA concentration testing, checkerboard assays with FICI analysis, time-kill assays, CFU recovery, and regrowth assays. Finally, biofilm susceptibility was primarily assessed using the XTT reduction assay, which measures metabolic activity rather than definitive fungal eradication. Accordingly, the reported MBEC values should be interpreted as XTT-derived metabolic inhibition thresholds rather than sterilization endpoints. Additional viability, imaging, and biomass assays are needed to determine whether these metabolic effects correspond to true fungal killing or structural biofilm disruption. The cytotoxicity and local tissue compatibility of 50 mg/mL TXA were not evaluated, and the mechanisms underlying TXA-antifungal interactions remain to be clarified in future studies.

## Conclusions

TXA modulated the apparent in vitro activity of representative antifungal agents against a single susceptible *C. albicans* reference strain in an antifungal class- and growth-state-dependent manner. Under planktonic conditions, TXA was associated with increased apparent potency of VRC and CAS, no detectable change in FLC activity, and reduced apparent potency of AMB. Under established biofilm conditions, TXA enhanced FLC-mediated metabolic suppression and improved CAS activity at submaximal exposures, whereas it attenuated the XTT-derived biofilm metabolic inhibition achieved by VRC and AMB. These findings suggest that TXA is not pharmacologically neutral in antifungal susceptibility testing and may differentially influence antifungal performance depending on the drug class and fungal growth state. Further studies using resistant and clinical *Candida* isolates, non-*albicans Candida* species, clinically relevant biofilm models, and complementary viability and mechanistic assays are required to determine whether these TXA-associated effects persist under conditions that more closely reflect fungal PJI in vivo.

## Data Availability

All data generated or analysed during this study are included in this published article. Additional raw data are available from the corresponding authors on reasonable request.

## Abbreviations

AMB: Amphotericin B
ATCC: American Type Culture Collection
*C. albicans*: *Candida albicans*
CAS: Caspofungin
CFU: Colony-forming units
CLSI: Clinical and Laboratory Standards Institute
FLC: Fluconazole
MBEC: Minimum biofilm eradication concentration
MBEC_50_: Minimum biofilm eradication concentration required for 50% metabolic inhibition
MBEC_90_: Minimum biofilm eradication concentration required for 90% metabolic inhibition
MIC: Minimum inhibitory concentration
OD600: Optical density at 600 nm
PJI: Periprosthetic joint infection
RPMI: Roswell Park Memorial Institute
SDA: Sabouraud dextrose agar
TXA: Tranexamic acid
VRC: Voriconazole
XTT: 2,3-bis(2-methoxy-4-nitro-5-sulfophenyl)-2 H-tetrazolium-5-carboxanilide
YPD: Yeast extract peptone dextrose
